# Serotonin receptor subtype-2B signaling is associated with interleukin-18-induced cardiomyoblast hypertrophy in vitro

**DOI:** 10.2478/abm-2022-0010

**Published:** 2022-04-29

**Authors:** Chao-Yi Chen, Jyh-Gang Leu, Kuan-Yu Lin, Chin-Yu Shih, Yao-Jen Liang

**Affiliations:** Graduate Institute of Applied Science and Engineering, Fu-Jen Catholic University, New Taipei City 242062, Taiwan; Department and Institute of Life Science, Fu-Jen Catholic University, New Taipei City 242062, Taiwan; Fu-Jen Catholic University School of Medicine, New Taipei City 242062, Taiwan; Division of Nephrology, Department of Internal Medicine, Shin Kong Wu Ho-Su Memorial Hospital, Taipei 111, Taiwan

**Keywords:** hypertrophy, interleukin-18, myocytes, cardiac, receptor, serotonin, 5-HT2B, signal transduction

## Abstract

**Background:**

In patients with heart failure, interleukin-18 (IL-18) levels increase in the circulatory system and injured myocardial tissue. Serotonin (5-hydroxytryptamine) receptors subtype 2B (HTR2B) play an essential role in cardiac function and development, and their overexpression in rats leads to myocardial hypertrophy. Epigallocatechin gallate (EGCG) is cardioprotective in myocardial ischemia–reperfusion injury in rats and can prevent pressure overload-mediated cardiac hypertrophy in vivo. Mice deficient in peroxisome proliferator-activated receptor delta (PPARδ) can have cardiac dysfunction, myocardial hypertrophy, and heart failure. Matrix metalloproteinases (MMPs) are possibly involved in cardiac remodeling. However, the relationship between IL-18 signaling, cardiac hypertrophy, and the molecular mechanisms involved remain to be fully elucidated.

**Objectives:**

To elucidate the relationship between HTR2B and IL-18-induced myocardial hypertrophy and examine the antihypertrophic effects of EGCG and PPARδ.

**Methods:**

We induced H9c2 cardiomyoblast hypertrophy with IL-18 in vitro and investigated the downstream signaling by real-time polymerase chain reaction (PCR) and western blotting. Hypertrophy was assessed by flow cytometry. We determined the effects of EGCG and PPARδ on IL-18-induced hypertrophic signaling via HTR2B-dependent mechanisms.

**Results:**

IL-18-induced H9c2 hypertrophy upregulated brain natriuretic peptide (BNP) protein and mRNA expression by inducing the expression of nuclear factor kappa-light-chain-enhancer of activated B cells (NF-κB), and the hypertrophy was attenuated by pretreatment with EGCG (20 μM) and L-165,041 (2 μM), a PPARδ agonist. IL-18 upregulated the expression of HTR2B, which was inhibited by pretreatment with EGCG and L-165,041. SB215505 (0.1 μM), a HTR2B antagonist and siRNA for HTR2B, attenuated H9c2 hypertrophy significantly. Inhibition of HTR2B also downregulated the expression of MMP-3 and MMP-9.

**Conclusions:**

IL-18 and HTR2B play critical roles in cardiomyoblast hypertrophy. EGCG and L-165,041 inhibit the expression of HTR2B and augment remodeling of H9c2 cardiomyoblasts, possibly mediated by MMP-3 and MMP-9.

Heart hypertrophy can cause cardiac failure and even death; consequently, cardiac remodeling is an important clinical problem [1]. An obvious change in morphology is a key characteristic of cardiac hypertrophy; the change is due mainly to cardiomyocyte enlargement, which causes the heart wall to thicken, and thus reduces the size of the ventricular chambers [2].

Interleukin-18 (IL-18)-induced ventricular cell hypertrophy induces the expression of atrial natriuretic peptide (ANP) mRNA and protein synthesis. IL-18 also induces the expression of GATA4 transcription factor in vitro, and activates protein kinase B (Akt) via a phosphatidylinositol 3-kinase (PI3K)–phosphoinositide-dependent kinase (PDK1) pathway [3, 4]. Activation of these pathways by IL-18 signaling may be related to cardiac hypertrophy. Clinically, higher serum IL-18 levels are associated with the occurrence of congestive heart failure, coronary artery disease, and myocardial ischemia, and IL-18 is found in symptomatic (unstable) atherosclerotic plaques [5, 6]. This suggests that chronic serum IL-18 elevation is associated with cardiac hypertrophy. Therefore, IL-18 is considered to play an important role in maladaptive myocardial hypertrophy [7]. The relationship between IL-18 signaling and cardiac hypertrophy remains to be fully elucidated. Here, we sought to investigate possible mechanisms of cardiomyocyte hypertrophy induced by IL-18.

Serotonin (5-hydroxytryptamine or 5-HT) receptor 2B (HTR2B) is a subtype of 5-HT receptor. HTR2B are expressed mainly in the embryonic development of mouse, rat, and adult human cardiovascular tissue [8]. 5-HT plays an important role in regulating heart development and function through HTR2B [9]. In the hearts of rats, overexpression of HTR2B induces mitochondrial hyperplasia, leading to myocardial hypertrophy [10]. 5-HT-induced hypertrophy is observed in adult rat left ventricular cell cardiomyocytes in vitro. After mechanical stress, increased HTR2B expression in cardiomyocytes leads to increased expression of brain natriuretic peptide (BNP), which is regulated through nuclear factor kappa-light-chain-enhancer of activated B cells (NF-κB) translocation [11]. Myocardial hypertrophy induced by isoproterenol and HTR2B plays a role in myocardial fibroblast signaling [12]. It seems clear that HTR2B plays an essential role in cardiac function and development. However, like the relationship between IL-18 signaling and cardiac hypertrophy, the role of HTR2B in cardiomyocyte hypertrophy remains to be fully elucidated. Manipulating HTR2B may provide a new approach to treatment of myocardial hypertrophy or heart failure.

Peroxisome proliferator-activated receptors (PPARs) are involved in the regulation of lipid metabolism and glucose homeostasis. In the cardiovascular system, they are involved in the regulation of cell growth. Mice deficient in PPARδ have been found to have cardiac dysfunction, myocardial hypertrophy, and heart failure, evidence that PPARδ plays a role in myocardial pathology [13]. Epigallocatechin gallate (EGCG), a polyphenol catechin component of green tea, can prevent the hypertrophy of vascular smooth muscle cells induced by angiotensin II by inhibiting c-Jun N-terminal kinases [14, 15]. These findings suggest that EGCG could regulate myocardial hypertrophy. HTR2 and monoamine oxidase A (MAO-A) contribute to H9c2 cardiomyoblast hypertrophy, and the MAO-A-dependent hypertrophic response required activation of extracellular signal-regulated kinases (ERKs) [16].

Here, we examined the effect of EGCG and a PPARδ agonist (L-165,041) on IL-18-induced H9c2 hypertrophy. In addition, we sought to elucidate the relationship between IL-18 and HTR2B-induced cardiomyoblast hypertrophy.

## Methods

### Cell culture and reagents

H9c2 myoblasts were obtained from the Bioresource Collection and Research Center (Hsinchu, Taiwan), and the detection and verification of the cardiomyoblast cell line was based on Kimes [17]. Cells were cultured in Dulbecco's modified Eagle's medium (Gibco) containing 10% fetal bovine serum (Gibco) and 1% antibiotic–antimycotic (Gibco), and were maintained in an incubator at 37 °C under a humidified atmosphere of 5% CO_2_ in air. Cardiomyoblasts in the experimental groups were pretreated with serotonin at 10 μM, EGCG (Sigma-Aldrich) at 20 μM, PPARδ agonist L-165,041 (Sigma-Aldrich) at 2 μM, or HTR2B antagonist SB215505 (Sigma-Aldrich) at 0.1 μM for 30 min, and were subsequently treated with IL-18 (ProSpec; Ness-Ziona, Israel) at 0.3 μg/mL for 18 h.

### Western blotting

Nuclear or total protein was extracted from cardiomyoblasts homogenized in cell lysis buffer (Pro-Prep; iNtRON Biotechnology; Gyeonggi-do, Korea). Protein concentrations were determined using a BCA Assay Kit (Thermo Fisher Scientific). We mixed 30 μg protein samples with sample buffer (150 μM NaCl, 1% Triton X-100), 0.5% sodium deoxycholate, and 0.1% sodium dodecyl sulfate (SDS), heated the mixture in a boiling water bath for 5 min, and separated the proteins by SDS–10% polyacrylamide gel electrophoresis (PAGE) under denaturing conditions (gel 10 cm × 8 cm × 1 mm; running voltage 80 V for 2 h; Hoefer SE260 Protein Electrophoresis Unit). Proteins in the gel were transferred (running voltage: 80 mA for 80 min; Hoefer TE70XP Semi-Dry Transfer) to PVDF membranes (Immobilon-P transfer membrane; Millipore), which were then soaked in methanol for 5 min. Nonspecific binding sites on the membranes were blocked with 5% skim milk in phosphate-buffered saline (PBS) containing 0.1% Tween 20 (Tween) for 60 min, and then incubated with anti-NIK (1:5,000 in PBS-Tween, Santa Cruz Biotechnology cat. No. sc-8417; Research Resource Identifier (RRID): AB_628021, https://scicrunch.org/resources), anti-NF-κB (1:5000, Santa Cruz Biotechnology cat. No. sc-8008; RRID: AB_628017), anti-BNP (1:5000, Santa Cruz Biotechnology cat. No. sc-271185; RRID: AB_10609757), anti-MMP-3 (1:5000, Santa Cruz Biotechnology cat. No. sc-21732; RRID: AB_627958), anti-MMP-9 (1:5000, Santa Cruz Biotechnology cat. No. sc-21733; RRID: AB_627959), anti-HTR2B (1:5000, Santa Cruz Biotechnology cat. No. sc-25647; RRID: AB_2124523), anti-C23 (1:5000, Santa Cruz Biotechnology cat. No. sc-8031; RRID: AB_670271), or anti-β-actin (1:5000, Santa Cruz Biotechnology cat. No. sc-47778; RRID: AB_626632) antibodies overnight at 4 °C. The membranes were washed with PBS-Tween and subsequently incubated with horseradish peroxidase-conjugated secondary antibody (anti-goat, 1:5000 in PBS-Tween, Santa Cruz Biotechnology cat. No. sc-2020; RRID: AB_631728; anti-rabbit, 1:5000, Santa Cruz Biotechnology cat. No. sc-2004; RRID: AB_631746, or anti-mouse, 1:5000, Santa Cruz Biotechnology cat. No. sc-2005, RRID: AB_631736) as appropriate for 1 h at room temperature. Signals were visualized by enhanced chemiluminescent detection.

### Real-time quantitative polymerase chain reaction (qPCR)

Total RNA was extracted from H9c2 using TRIzol and reverse-transcribed to cDNA. Amplification reactions were performed in volumes of 50 μL containing reaction buffer, 25 μL Smart Quant Green Master Mix with dUTP (Protech Technology Enterprise; Taipei, Taiwan), 4 mM MgCl_2_, 50 ng template cDNA, and 1 μM target primer. Primer sequences were as follows: BNP (forward: 5′-AGGCAGAGTCAGAAGCCAGAGT-3′; reverse: 5′-CTTAGGTCTCAAGACAGCGCCT-3′), HTR2B (forward: 5′-GCCTTCTTCACACCTCTTGC-3′; reverse: 5′-GTCCTTTCGAGAACCATCCA-3′), or β-actin (forward: 5′-ATGCCAACACAGTGCTGTCTGG-3′; reverse: 5′-TACTCCTGCTTGCTGATCCACAT-3′). The amplification was detected using a StepOne Real-Time PCR System (Thermo Fisher Scientific). The initial denaturizing phase was 10 min at 95 °C followed by an amplification phase as described following: denaturation at 95 °C for 15 s; annealing and elongation at 60 °C for 60 s, 40 cycles; the final elongation step was 95 °C for 15 s, 60 °C for 60 s, and 95 °C for 15 s. The BNP and HTR2B Ct values were obtained and the relative fold change in gene expression was calculated as fold changes in gene expression after normalizing to β-actin using the formula 2^−ΔΔCt^.

### Cardiomyoblast hypertrophy analysis

H9c2 were seeded (1 × 10^6^ cells) in 6 cm diameter dishes. The cells were pretreated with EGCG, L-164,041, or SB215505 for 30 min, and subsequently treated with IL-18 for 18 h. After treatment, the cells were harvested, washed, and resuspended in PBS. Cells were maintained on ice for 30 min. At least 10^4^ cells were collected and analyzed using a CytoFLEX LX Flow Cytometer (Beckman Coulter). The size of cells was determined using CytExpert version 2.2 (Beckman Coulter).

### RNA interference

For HTR2B siRNA (Dharmacon) transfection, H9c2 were seeded (1 × 10^6^ cells) in 6 cm diameter dishes before transfection. H9c2 were transfected with 2 μg HTR2B siRNA using Lipocurax siRNA transfection reagents (Ambo Life; Taoyuan, Taiwan), and then cultured in an incubator at 37 °C under a humidified atmosphere of 5% CO_2_ in air for 18 h. The transfected cells were treated with IL-18 for 18 h. Protein samples were analyzed by western blotting.

### Statistical analysis

All data were analyzed using IBM SPSS Statistics for Windows (version 24). The data are normalized and expressed as mean ± standard error of mean (SEM) and individual data. Data between 2 groups were compared using a Mann–Whiney *U* test, and a Kruskal–Wallis test was used to compare data from 3 or more independent groups. Differences between group data with *P* < 0.05 were considered significant.

## Results

### EGCG and PPARδ agonist (L-165,041) attenuated IL-18-induced inflammation in H9c2 cardiomyoblasts

**Figure 1:**
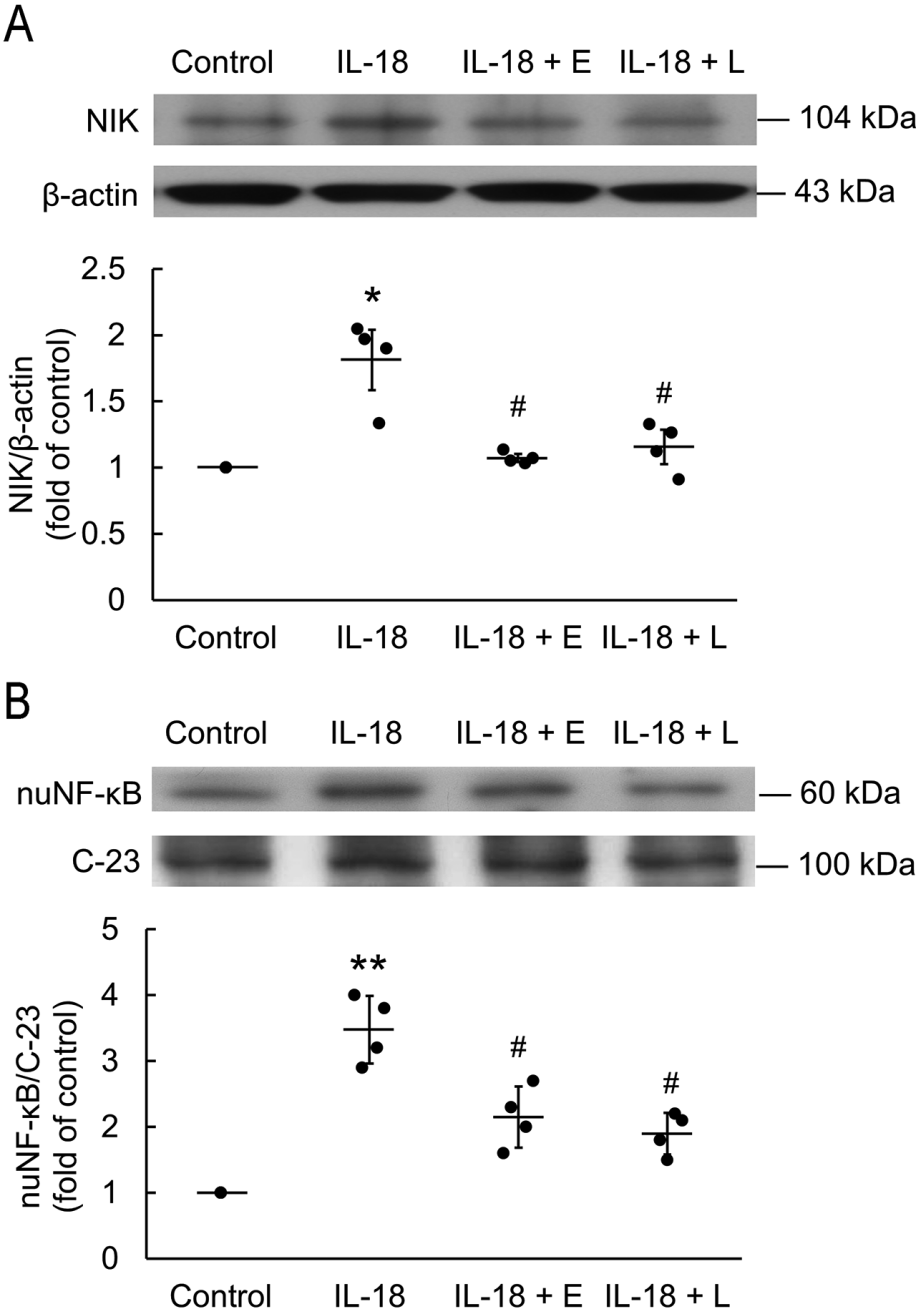
Pretreatment with 20 mM ECGC and 2 mM L-165,041 attenuated IL-18-induced inflammatory markers in H9c2 cardiomyoblasts. (**A**) NIK production significantly was inhibited by EGCG or L-165,041 (n = 4). (**B**) EGCG and L-165,041 significantly attenuated IL-18-induced nuNF-κB upregulation (n = 4). The data are normalized and shown as individual data (solid circles) and as mean (horizontal lines) ± SEM (error bars). Differences between groups were assessed using a Kruskal–Wallis test. *P < 0.05 and **P < 0.01 when compared with control. #*P* < 0.05 when compared with IL-18 alone. ECGC (E), epigallocatechin gallate; IL-18, interleukin-18; L-165,041 (L), peroxisome proliferator-activated receptor delta (PPARd) agonist; NIK, nuclear factor kappa-light-chain-enhancer of activated B cells (NF-κB) inducing kinase; nuNF-κB, nuclear NF-κB; SEM: standard error of mean.

H9c2 were incubated with 0.3 μg/mL IL-18 for 18 h. IL-18 significantly increased the expression of NF-κB inducing kinase (NIK) in the H9c2 (**Figure 1A**). Pretreatment of H9c2 with EGCG or L-165,041 significantly attenuated the NIK expression otherwise significantly upregulated by incubation with IL-18 for 18 h. Both EGCG and L-165,041 significantly attenuated the nuclear NF-κB (nuNF-κB) expression otherwise significantly upregulated by incubation with IL-18 after 18 h (**Figure 1B**).

### IL-18 upregulates BNP mRNA and protein in H9c2 cardiomyoblasts

**Figure 2:**
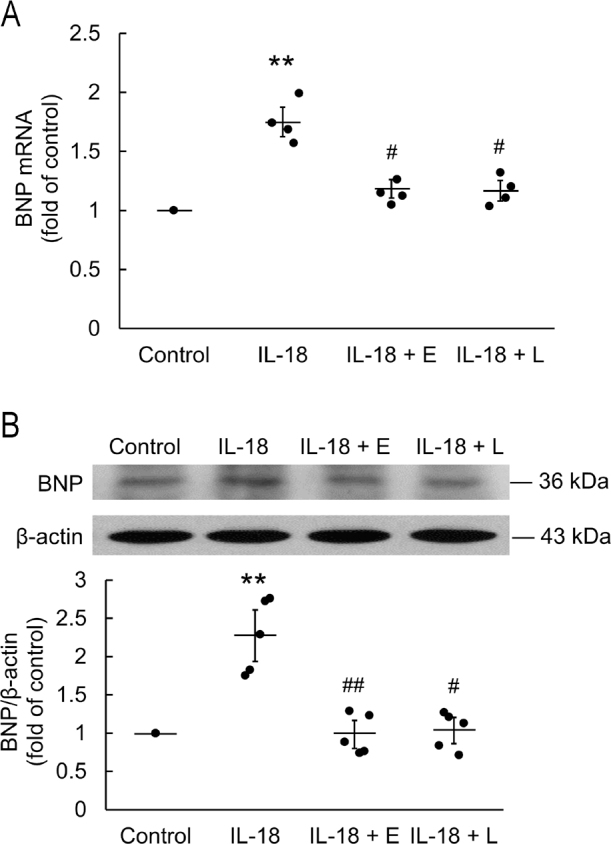
Effects of 20 mM ECGC and 2 mM L-165,041 treatment on IL-18-induced BNP mRNA and protein expressions in H9c2 cardiomyoblasts. Treatment with IL-18 significantly increased the BNP (n = 4) (**A**) mRNA and (**B**) protein expression (n = 5). The data are normalized and shown as individual data (solid circles) and as mean (horizontal lines) ± SEM (error bars). Differences between groups were assessed using a Kruskal–Wallis test. **P < 0.01 when compared with control. #*P* < 0.05 and ##*P* < 0.01 when compared with IL-18. BNP, brain natriuretic peptide; ECGC (E), epigallocatechin gallate; IL-18, interleukin-18; L-165,041 (L), peroxisome proliferator-activated receptor delta (PPARd) agonist; SEM: standard error of mean.

Real-time qPCR showed that incubation with IL-18 for 18 h significantly upregulated BNP mRNA (*P* < 0.01) and pretreatment with EGCG or L-165,041 significantly attenuated the expression of BNP otherwise significantly upregulated by incubation with IL-18 (**Figure 2A**). Western blotting showed that the significant upregulation of BNP after incubation with IL-18 for 18 h was attenuated by pretreatment with EGCG or L-165,041 (*P* < 0.01 and *P* < 0.05, respectively; **Figure 2B**).

### IL-18 upregulates HTR2B mRNA and protein in H9c2 cardiomyoblasts

**Figure 3:**
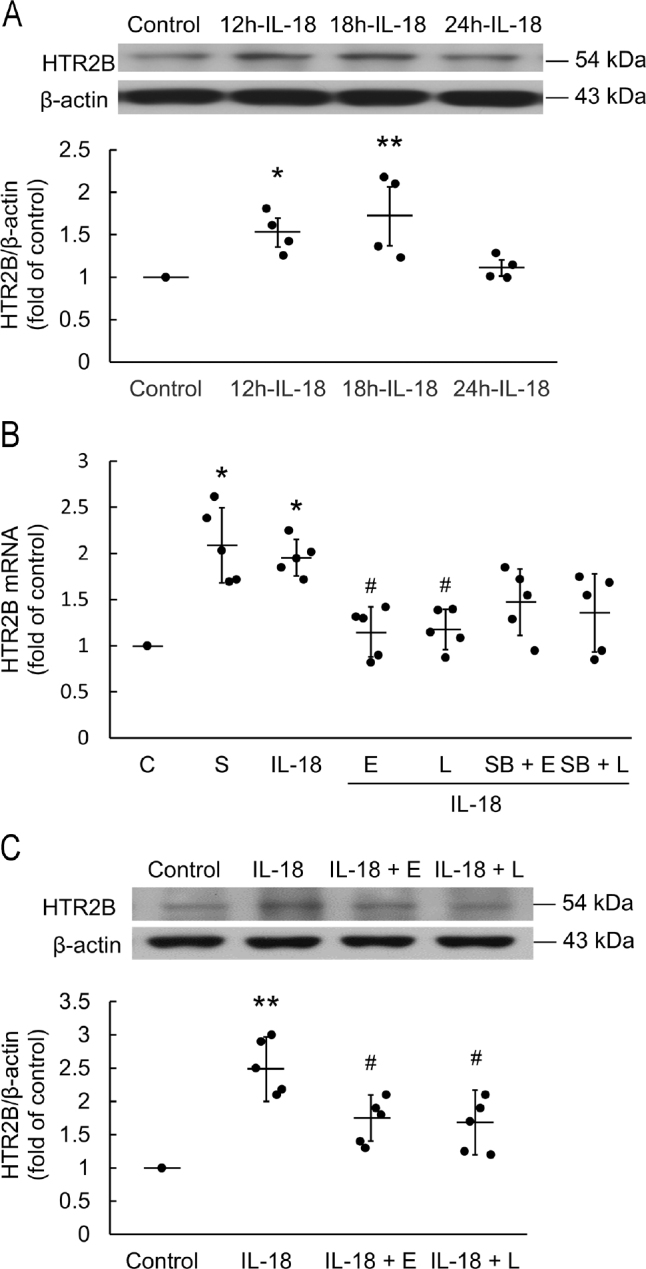
Pretreatment with 20 μM EGCG and 2 μM L-165,041 inhibited IL-18-induced HTR2B upregulation. (**A**) H9c2 cardiomyoblasts treated with IL-18 for up to 24 h, IL-18-induced HTR2B protein expression. Total protein was extracted and analyzed by western blotting for HTR2B protein expression (n = 4). (**B**) Serotonin (S) alone (10 μM) significantly increased the expression of HTR2B mRNA. H9c2 were treated IL-18 for 18 h. Pretreatment with EGCG and L-165,041 inhibited IL-18-induced HTR2B mRNA upregulation (n = 5) and (**C**) HTR2B protein expression (n = 5). The data are normalized and shown as individual data (solid circles) and as mean (horizontal lines) ± SEM (error bars). Differences between groups were assessed using a Kruskal–Wallis test. *P < 0.05 and **P < 0.01 when compared with control. #*P* < 0.05 when compared to IL-18 treatment alone. HTR2B, 5-hydroxytryptamine receptor 2B; ECGC (E), epigallocatechin gallate; IL-18, Interleukin-18; L-165,041 (L), peroxisome proliferator-activated receptor delta (PPARδ) agonist; SB215505 (SB), HTR2B antagonist; serotonin, 5-hydroxytryptamine.

H9c2 were incubated with 0.3 μg/mL IL-18 for various times. IL-18 significantly increased the expression of HTR2B protein in H9c2 after 12 h and 18 h (*P* = 0.011 and *P* = 0.007, respectively), but the expression had returned to control levels within 24 h (**Figure 3A**). Serotonin alone significantly increased HTR2B mRNA expression (*P* = 0.03). Both EGCG and L-165,041 significantly attenuated the HTR2B mRNA upregulation by IL-18 after 18 h (*P* = 0.012 and *P* = 0.025, respectively). However, after pretreatment with SB215505, the attenuation effect was diminished (**Figure 3B**). Consistent with this finding, HTR2B protein was significantly upregulated by incubation with IL-18 for 18 h, as found by western blotting (*P* < 0.01). Pretreatment with EGCG and L-165,041 significantly attenuated the increased HTR2B protein expression seen after incubation with IL-18 (**Figure 3C**).

### Relationship between IL-18-induced H9c2 cardiomyoblast hypertrophy and HTR2B

**Figure 4:**
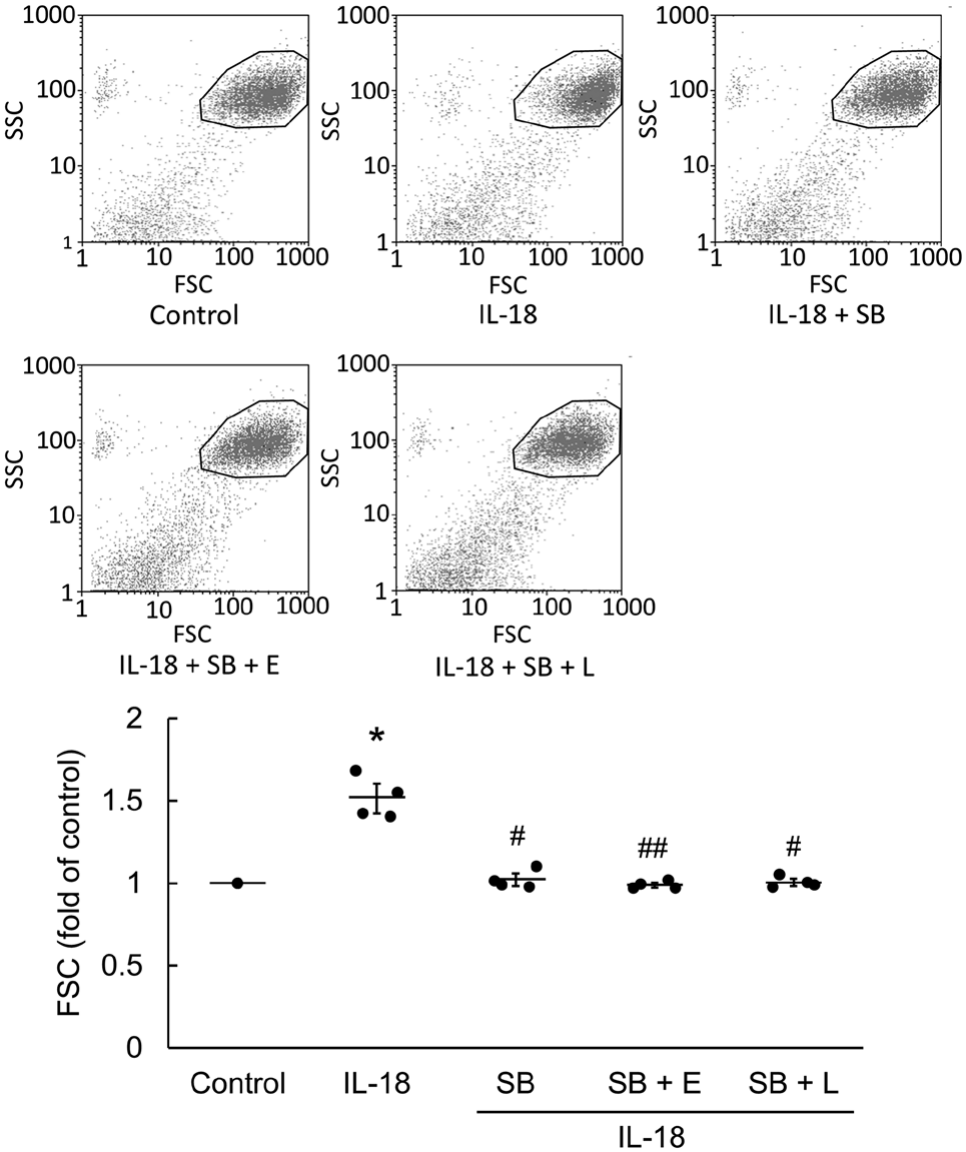
Pretreatment with 0.1 mM SB215505, 20 mM EGCG, and 2 mM L-165,041 inhibited IL-18-induced H9c2 hypertrophy. H9c2 hypertrophy was inhibited by SB215505, EGCG, and L-165,041, as analyzed by flow cytometry. FSC reflects the relative size of cells. The relative average size for control was 282; IL-18 was 354; IL-18 + SB was 304; IL-18 + SB + EGCG was 272; and IL-18 + SB + L-165,041 was 273 (n = 4). The data are normalized and shown as individual data (solid circles) and as mean (horizontal lines) ± SEM (error bars). Differences between groups were assessed using a Kruskal–Wallis test. *P < 0.05 when compared with control. #*P* < 0.05 and ##*P* < 0.01 when compared with IL-18. ECGC (E), epigallocatechin gallate; FSC, forward scatter; IL-18, interleukin-18; L-165,041 (L), peroxisome proliferator-activated receptor delta (PPARd) agonist; SB215505 (SB), HTR2B antagonist; SEM, standard error of mean; SSC, side scatter. HTR2B, 5-hydroxytryptamine receptor 2B.

**Figure 5:**
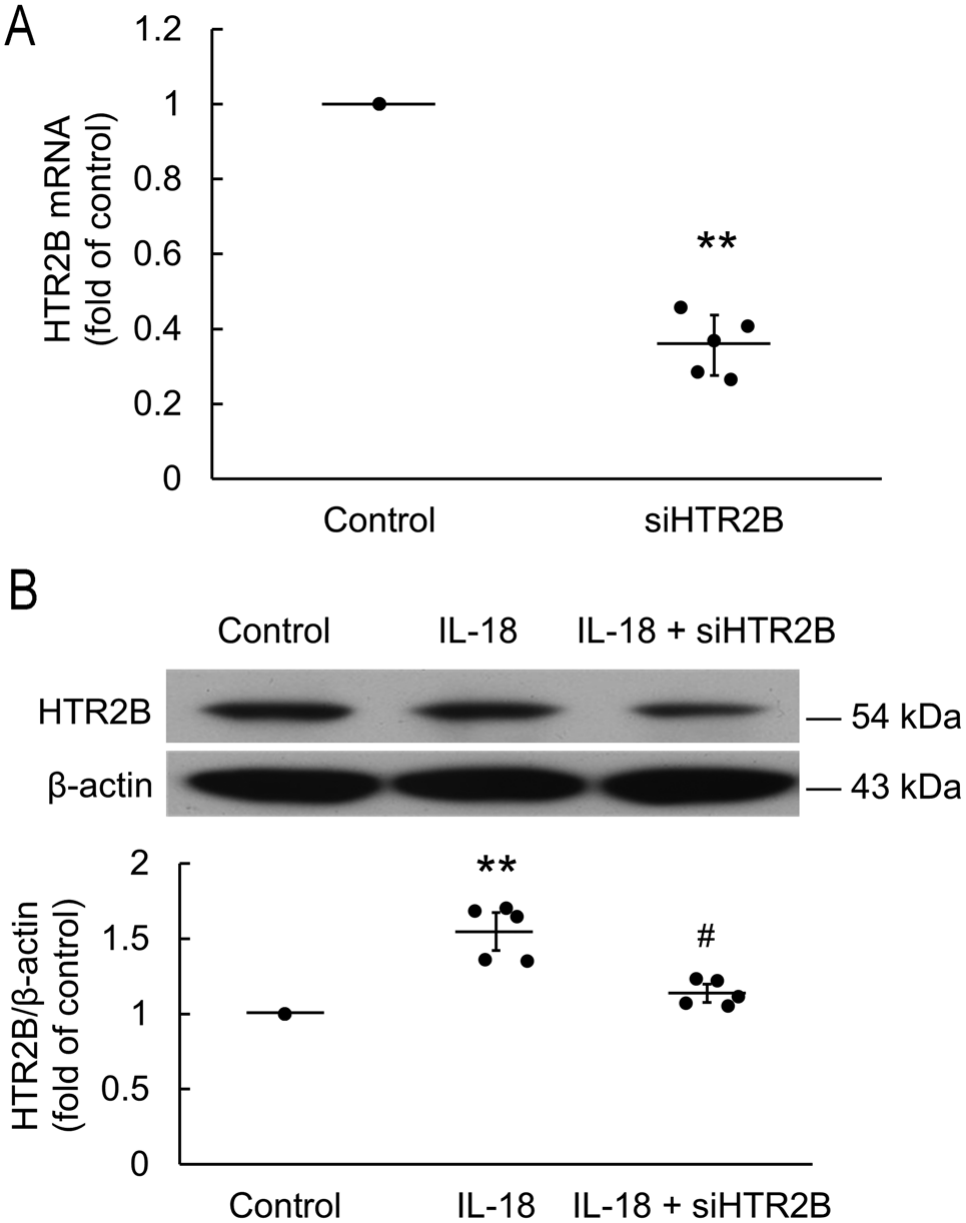
siHTR2B significantly attenuated IL-18-induced HTR2B expression. The data are normalized and shown as individual data (solid circles) and as mean (horizontal lines) ± SEM (error bars). (**A**) Pretreatment with siHTR2B significantly decreased HTR2B mRNA expression (n = 5). Differences from control were assessed using a Mann–Whitney *U* test. (**B**) Total protein was extracted and analyzed by western blotting for HTR2B protein expression (n = 5). Pretreatment with siHTR2B significantly inhibited IL-18-induced HTR2B protein expression. Differences between groups were assessed using a Kruskal–Wallis test. **P < 0.01 when compared with control. #*P* < 0.05 when compared with IL-18. HTR2B, 5-hydroxytryptamine receptor 2B; IL-18, interleukin-18; SEM, standard error of mean.

Incubation of H9c2 with IL-18 increased the size of the cardiomyoblasts after 18 h (*P* = 0.031). Pretreatment with HTR2B antagonist (SB215505) significantly inhibited IL-18-induced cell hypertrophy (*P* = 0.031). EGCG and L-165,041 did not augment the effect of SB215505 (**Figure 4**). siRNA alone acted as a negative control (*P* < 0.01; **Figure 5A**). Incubation of H9c2 with IL-18 for 18 h upregulated HTR2B protein expression (*P* < 0.01), and siHTR2B treatment significantly inhibited the expression of HTR2B (*P* = 0.042; **Figure 5B**).

### IL-18 induces H9c2 cardiomyoblast hypertrophy through the matrix metalloproteinase-3 (MMP-3) and MMP-9

**Figure 6:**
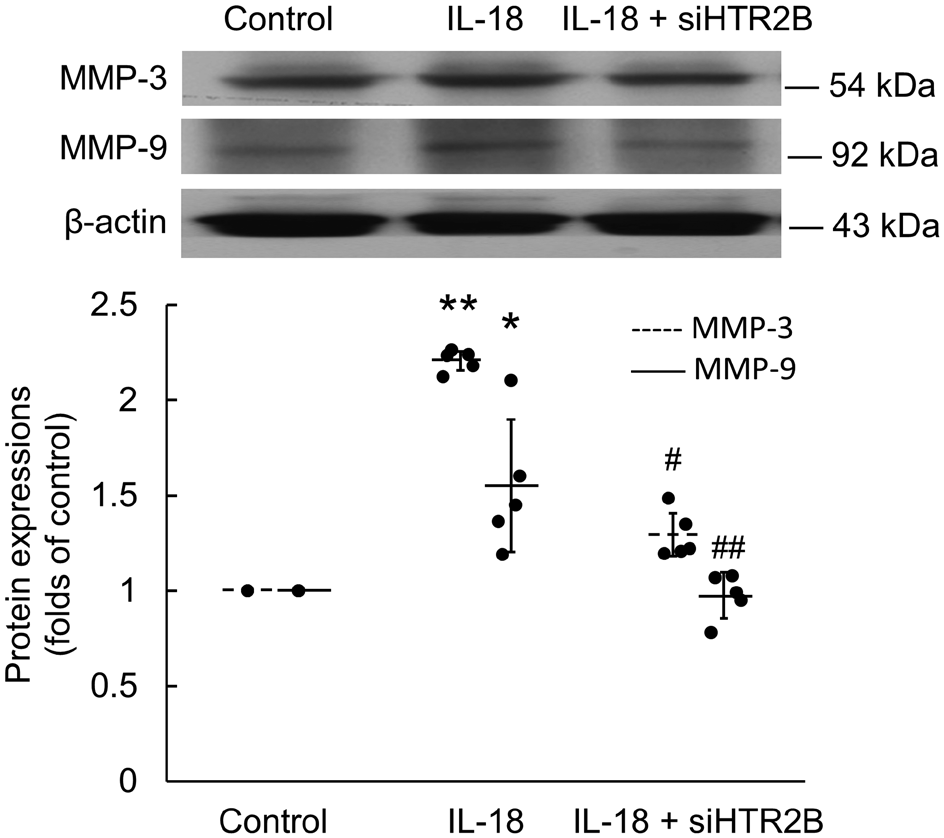
Pretreatment with siHTR2B significantly attenuated IL-18-induced MMP-3 and MMP-9 expression. Total protein was extracted and analyzed by western blotting for MMP-3 and MMP-9 expression (n = 5). The data are normalized and shown as individual data (solid circles) and as mean (horizontal lines; dashed, MMP-3; solid, MMP-9) ± SEM (error bars). Differences between groups were assessed using a Kruskal–Wallis test. *P < 0.05 and **P < 0.01 when compared with control. #*P* < 0.05 and ##*P* < 0.01 when compared with IL-18 alone. HTR2B, 5-hydroxytryptamine receptor 2B; IL-18, interleukin-18; MMP-3, matrix metalloproteinase-3; MMP-9, matrix metalloproteinase-9; SEM, standard error of mean.

Incubation of H9c2 with IL-18 for 18 h significantly upregulated their expression of MMP-3 protein (*P* < 0.01). Pretreatment with siHTR2B attenuated the IL-18-induced MMP-3 protein expression (*P* = 0.034; **Figure 6**). Incubation of H9c2 with IL-18 for 18 h significantly upregulated the expression of MMP-9 protein (*P* = 0.012), and pretreatment with siHTR2B significantly attenuated the IL-18-induced MMP-9 protein expression (*P* = 0.004; **Figure 6**).

## Discussion

The present results confirm that the mechanism of IL- 18- induced H9c2 cardiomyoblast hypertrophy involves regulation of NIK, NF-κB, and BNP pathways, and the expression of HTR2B. HTR2B are considered to play a crucial role and chronic activation of HTR2B might contribute to cardiomyocyte hypertrophy [18], which results from its downstream pathways. HTR2B are considered to play an integral role in IL-18-induced H9c2 hypertrophy and remodeling of myocardial cells. To determine the effect of the PPARδ agonist, we chose H9c2, which have high PPAR expression [19]. However, further experiments will need to be conducted using primary cardiomyocyte cultures and in vivo to confirm our findings.

There are various causes of cardiac hypertrophy, and one of them is IL-18 acting as a cellular prehypertrophic cytokine. In previous studies, IL-18 was found to act via PI3K/PDK1/Akt/GATA4-induced cardiac hypertrophy and play an important role in cardiac remodeling and heart failure. IL-18 also induced ANP gene transcription to cause hypertrophy [20]. IL-18-induced human cardiac microvascular endothelial cell death via a NF-κB-dependent signaling pathway [21]. Inhibition of NF-κB in vivo was sufficient to attenuate angiotensin II- and isoproterenol-induced hypertrophy of cardiomyocytes [22]. Together these results indicate that NF-κB plays an important role in cardiac hypertrophy.

In the present study, western blotting showed that IL-18 upregulated NIK and nuNF-κB in an inflammatory NIK/NF-κB signaling pathway in H9c2. In previous studies of cultured cardiomyocytes, the increase in the size of cardiomyocytes subjected to a hypertrophic stimulus was associated with increased expression of natriuretic peptides at both the mRNA and protein levels [23]. Previous studies have found that IL-18 induces expression of NF-κB [24], which impairs cardiac function, and was dependent on myeloid differentiation 88 (MyD88) → interleukin 1 receptor (IL-1R) associated kinase (IRAK) → TNF receptor-associated factor 6 (TRAF6) → PI3K → Akt → induced IκB kinase (IKK) → NF-κB inducing the expression of fibronectin [3, 4, 21], and further mediated myocardial hypertrophy. In the present study, we showed BNP mRNA and protein expression were significantly upregulated after IL-18 stimulation of H9c2 cardiomyoblasts. IL-18 caused NF-κB-induced hypertrophy of H9c2 and increased BNP expression. We observed the expression of NIK, NF-κB, and BNP individually, and found an increasing trend after 18 h of IL-18 stimulation; therefore, it is assumed that IL-18 → NIK → NF-κB → BNP further affected the H9c2 hypertrophy. We propose this hypertrophic signaling pathway in H9c2 cardiomyoblasts.

In previous studies, EGCG blocked the activation of NF-κB by inhibiting the activity of IKK [25]. In addition, ventricular hypertrophy induced by transverse abdominal aortic constriction in rats with hypertension could be attenuated by EGCG, which inhibits the activation of mitogen-activated protein kinase (MAPK) [26]. We found that EGCG could reduce IL-18-induced NF-κB activation, which subsequently reduced the expression of BNP, and reduced cardiomyoblast hypertrophy as seen by flow cytometry. Previous studies have highlighted that lipopolysaccharide-induced myocardial hypertrophy was associated with a decrease in the oxidation of fatty acids and an increase in the utilization of glucose [27]. Myocardial hypertrophy can be caused by activation of NF-κB and reduced regulation of fatty acid oxidation. In the present study, we used L-165,041 to activate PPARδ and investigated whether this reduced myocardial hypertrophy. Activation of PPARδ in H9c2 cardiomyoblasts activates expression of PPARα target genes involved in fatty acid utilization in cardiaomyocytes [28]. Therefore, H9c2 cardiomyoblasts were selected for the experiments to obtain results specific to the PPARδ expression. We found that the addition of L-165,041 also inhibited the activation of NF-κB in IL-18-induced H9c2 hypertrophy.

HTR2B are important regulators of heart development and function [8, 9]. Overexpressing the 5-HT Gq-coupled HTR2B induces mitochondrial proliferation and myocardial hypertrophy in rats [10]. Moreover, HTR2B deficiency can cause dilated cardiomyopathy or myofibrillar breakdown [29]. In addition to the effects of HTR2B upregulation in cardiomyocytes, the expression of BNP is regulated through mechanical stress enhanced by HTR2B, which is mainly effected by NF-κB [11]. Therefore, for cardiomyopathy caused by hypertension, reducing the expression of HTR2B may provide a new approach to treatment of myocardial hypertrophy and consequent avoidance of this cause of heart failure. Under the stimulation of IL-18, activation of HTR2B might be directly or indirectly affected to activate the expression of NF-κB, which leads to an upregulation of BNP, and consequently, H9c2 hypertrophy. Therefore, HTR2B might play an important key role in the process of IL-18-induced myocardial hypertrophy. We used flow cytometry to determine whether pretreatment with SB215505 blunted the IL-18-induced hypertrophy of H9c2 and found that SB215505 attenuated the cell hypertrophy significantly. Therefore, we confirmed that HTR2B plays a critical role in the activation of NF-κB and BNP, and thus plays an integral role in IL-18-induced H9c2 cardiomyoblast hypertrophy. In previous studies, IL-18 binding protein (IL-18BP) acted as an antagonist of IL-18 to prevent some of the inflammatory responses induced by IL-18, and reverse damage to the myocardium [30]. In the present study, we found IL-18 upregulated HTR2B and played a critical role in myocardial hypertrophy. We consider myocardial hypertrophy and remodeling are two different concepts, and that inhibiting hypertrophy will lead to a compensatory effect of remodeling.

Here, we found that EGCG and L-165,041 attenuated the upregulation of IL-18-induced HTR2B expression by inhibiting HTR2B signaling. By pretreatment with the HTR2B antagonist SB215505, which inhibits the downstream signaling of HTR2B, the effect of EGCG and L-165,041 on inhibiting the upregulation of HTR2B was not augmented or diminished. This suggests that EGCG, L-165,041, and SB215505 may affect the same signaling pathway. Because SB215505 may not have absolute specificity for HTR2B, particularly at the concentration used (0.1 μM), we also inhibited HTR2B with siRNA to provide a control. We found that in the H9c2 stimulated with IL-18 for 18 h and pretreated with siHTR2B, the expression of MMP-3 and MMP-9 was decreased significantly. Neither EGCG nor L-165,041 augmented this effect, possibly because EGCG and L-165,041 downregulate the expression of MMP-3 and MMP-9 through a HTR2B-dependent pathway.

## Conclusions

IL-18 and HTR2B play critical roles in cardiomyoblast hypertrophy. EGCG and L-165,041 inhibit the expression of HTR2B and augment remodeling of H9c2 cardiomyoblasts, possibly mediated by MMP-3 and MMP-9. A new approach to treatment of myocardial hypertrophy and avoidance of consequent heart failure by targeting HTR2B seems plausible.
